# Cognitive Models in Cybersecurity: Learning From Expert Analysts and Predicting Attacker Behavior

**DOI:** 10.3389/fpsyg.2020.01049

**Published:** 2020-06-16

**Authors:** Vladislav D. Veksler, Norbou Buchler, Claire G. LaFleur, Michael S. Yu, Christian Lebiere, Cleotilde Gonzalez

**Affiliations:** ^1^DCS Corporation, U.S. Army Data & Analysis Center, Aberdeen Proving Ground, MD, United States; ^2^U.S. Army Data & Analysis Center, Aberdeen Proving Ground, MD, United States; ^3^Department of Psychology, Carnegie Mellon University, Pittsburgh, PA, United States

**Keywords:** cyber-security, cognitive modeling, behavioral simulations, deep learning, reinforcement learning, decision support, XAI (eXplainable Artificial Intelligence), human-agent teaming

## Abstract

Cybersecurity stands to benefit greatly from models able to generate predictions of attacker and defender behavior. On the defender side, there is promising research suggesting that Symbolic Deep Learning (SDL) may be employed to automatically construct cognitive models of expert behavior based on small samples of expert decisions. Such models could then be employed to provide decision support for non-expert users in the form of explainable expert-based suggestions. On the attacker side, there is promising research suggesting that model-tracing with dynamic parameter fitting may be used to automatically construct models during live attack scenarios, and to predict individual attacker preferences. Predicted attacker preferences could then be exploited for mitigating risk of successful attacks. In this paper we examine how these two cognitive modeling approaches may be useful for cybersecurity professionals via two human experiments. In the first experiment participants play the role of cyber analysts performing a task based on Intrusion Detection System alert elevation. Experiment results and analysis reveal that SDL can help to reduce missed threats by 25%. In the second experiment participants play the role of attackers picking among four attack strategies. Experiment results and analysis reveal that model-tracing with dynamic parameter fitting can be used to predict (and exploit) most attackers' preferences 40−70% of the time. We conclude that studies and models of human cognition are highly valuable for advancing cybersecurity.

## 1. Introduction

The field of cybersecurity has as much to do with human agency as it does with computer network integrity. However, while computer network technology changes rapidly on a regular basis, human learning and decision mechanisms do not. This being the case, research focused on cognitive science may provide the needed breakthrough capabilities for long-term network security and a greater return on investment than efforts chasing the latest software vulnerabilities. Cognitive science, and cognitive modeling in particular show a great promise for the field of cybersecurity (Veksler et al., 2018).

The goal of this paper is to provide examples of how computational models of human cognition may be employed to predict human preferences and behavior in cybersecurity. This paper focuses on two specific examples of the use of cognitive modeling in the context of cybersecurity. On the defender side, we aim to construct cognitive models of cyber analysts working with Intrusion Detection Systems (IDS) based on expert behavior, and employ these models to provide suggestions to non-expert analysts. On the attacker side, we aim to construct models of individual attacker decision biases, and employ these models to reduce the risk of successful attacks.

IDS software provides analysts with aggregated logs of network-activity alert records, where each record includes a number of threat-relevant features, and the job of a cyber analyst is to either elevate an alert as a potential threat, or to dismiss it as a false alarm. Predicting expert cyber analyst behavior in such a domain presents some challenges (Gonzalez et al., 2014). Traditional approaches in Computer Science routinely employ some Machine Learning (ML) classifier, training it on expert decisions with alert record features being classifier inputs, and the threat/no-threat classification being the output. Deep Learning (DL) methodology in particular has gained much acclaim in the recent decade as a successful technology for classifying large noisy complex data. The problem is that the availability of labeled cyber expert decision data is fairly sparse, often comprising just a few dozen or hundreds of examples (whereas DL requires training data with thousands or millions of examples). Additionally, DL-based recommendations are not easily explainable, and thus may not be well-suited for decision-aid software. Symbolic Deep Learning (SDL) may be a better approach for constructing models of expert behavior (Veksler and Buchler, 2019). The advantage of this approach is that it addresses the challenge of developing flexible and explainable models of cognition and behavior based on small samples of data.

Attacker-defender dynamics are often modeled in terms of Game Theory (GT). Game-theoretic approaches are useful for determining optimal mixes of strategies for leaving an attacker without a preferred strategy of their own. Moreover, GT-based defense algorithms have been successfully applied in many real-world security scenarios, including airport security, coast guard, police, and anti-poaching (animal preservation) efforts (Tambe et al., 2014). Veksler and Buchler (2016) and Cranford et al. (2019) argue that cognitive modeling techniques, and more specifically model-tracing^1^ and dynamic parameter fitting, may be used to track individual attacker preferences in real time, providing fairly high improvements over normative GT approaches in reducing the potential for successful attacks.

The rest of this paper presents two experiments with respective simulations and analyses, specifically aimed at examining the two uses of cognitive modeling in cybersecurity described above. In the first experiment participants play the role of cyber analysts performing a task based on IDS alert elevation. The SDL-based cognitive models are trained on data from top-performing participants. Results from all other participants are examined for degree of potential reduction in missed threats based on the trained SDL models. In the second experiment participants play the role of attackers picking among four attack strategies, and playing against defenders based on either normative game theory or against adaptive cognitive models using model-tracing with dynamic parameter fitting. Results from this experiment are analyzed for the degree to which human attacker preferences can be predicted and exploited.

## 2. Constructing Cognitive Models of Expert Analysts

A large subset of cybersecurity professionals are analysts working with Intrusion Detection Systems (IDS). This is often a grueling job requiring constant real-time monitoring of incoming network alerts for 12 hours straight (panama shifts). Employee turnover in these jobs is very high, in part because panama shifts are often incompatible with human health, mental health, and family life (Stimpfel et al., 2012; Oltsik, 2019). The job requires each analyst to sift through incoming alerts, picking out which alerts are worth elevating, and which are false alarms (D'Amico and Whitley, 2008). There is no way to train someone for this job via standard schooling, because every network has its own particularities, so all training is on-the-job training, even for experienced IDS professionals. In other words, this is a field where employees take a long time to gain expertise, and leave fairly soon after gaining said expertise.

Just like in the medical industry, the largest number of errors for IDS analysts happens after shift changes (which is the reason why panama shifts are the industry standard—to minimize the number of shift changes) (Friesen et al., 2008). After an employee spends 12 hours gaining expertise for the context of that day's alerts, they go home and leave the job to someone who is now missing all of the context needed for correct alert identification. The prescribed operating procedure is for each analyst to leave notes for the next shift, and to read notes left by analysts working the prior shift. However, based on our interviews with cyber-analysts, we found out that this rarely happens. Some analysts are better at taking and leaving notes than others, but there is always information lost between shifts.

A useful tool one might design for cyber-analysts would be a decision-aid that could highlight the alerts that an expert might elevate (whether we are talking about long-term expertise of veteran professionals, or more localized expertise relating to the state of recent network activity). This may be useful to enable new employees to see potential decisions of veteran employees, or it may be useful to enable analysts starting a shift to see potential decisions of analysts from the prior shift. In either case what is required is to train a model on contextualized expert decision-making and use it to predict future alert elevation.

In Machine Learning terms, the problem may be framed as a supervised classification problem where a model is trained on expert behavior, and its predictions are used for suggestions. Deep Learning in particular has garnered much attention over the last decade as being a highly successful ML technique for classification of complex and noisy data (e.g., Rusk, 2015). However, DL requires much larger training sets than are available in the context of expert analysts. Moreover, DL approaches are largely unexplainable and prone to catastrophic interference. That is, (1) there is no way for an analyst to ask “why” a specific alert was highlighted, and (2) updating the model with new expert decision data could cause the model to “forget” prior learned classifications.

Veksler and Buchler (2019) propose Symbolic Deep Learning (SDL) as an alternative approach to constructing user models. A symbolic version of deep learning is promising in that this method is capable of building classifiers from a small number of examples (Dutra et al., 2017; Zhang and Sornette, 2017; d'Avila Garcez et al., 2018). In this way, SDL learning efficiency is more akin to that of humans, and SDL is much more appropriate for creating models from individual or small-group data than DL. Whereas, DL builds up a black-box model of behavior, SDL builds up an explainable model of expert cognition—an expandable hierarchical memory network based on expert experiences and decisions.

**Figure 1:**
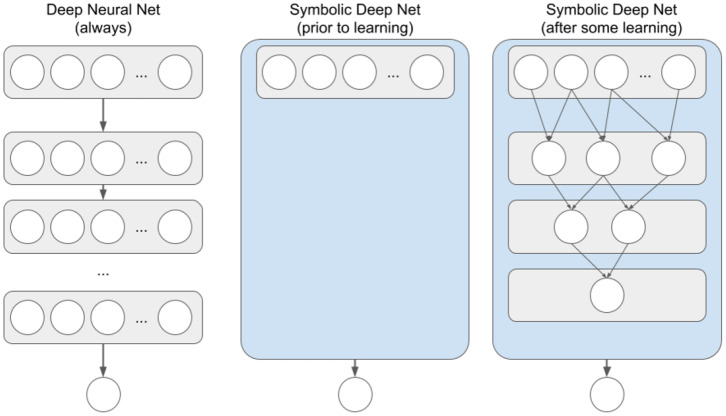
Traditional Deep Neural Net and Symbolic Deep Net structures for networks with a single output node. Each circle represents a network node. Top row of each net represents the input layer, bottom row is output nodes. Thicker arrows going from a node container to a node or another container represent a fully interconnected vector/matrix of weighted feed-forward links. Thinner arrows between nodes represent symbolic (i.e., not weighted) feed-forward links.

More specifically, a traditional deep neural network has a pre-specified number of layers, with a pre-specified number of nodes in each layer, and with nodes of each pair of successive layers being fully interconnected via weighted links (see Figure 1, left). As the network learns, the weights on these links change, but at no point can one look at those links and comprehend what exactly the network has learned. Because all knowledge is distributed among the links, the network has to be large enough to be able to learn a given problem, and thus requires thousands or millions of iterations to learn even simple input-output mapping. Symbolic deep nets, on the other hand, start with no nodes between input and output layers, and learn these nodes based on perceived input node co-occurrences (see Figure 1, middle and right). These deep nodes are essentially combinations of input features (a.k.a., chunks or configural cues), and in the case of modeling human memory, deeper chunks are taken to represent deeper domain expertise (Feigenbaum and Simon, 1984; Gobet, 1998). Due to the symbolic nature of chunk-based hierarchical memory, one can look at the learned chunks at any time so as to gain insight into what the network has learned. Because of the nature of chunk learning (one-shot learning), simple feature combinations can be learned quickly, enabling symbolic nets to learn at speeds on par with human learning—from just a few examples, rather than tens of thousands.

The major hurdle for symbolic deep models of memory has been a combinatoric explosion of memory. For example, the configural-cue model of memory (Gluck and Bower, 1988) creates a configural node (i.e., chunk) for every unique set of potential inputs, thus creating a maximum of (*k*+1)^*n*^−1 memory chunks, where *n* is the number of input dimensions and *k* is the number of possible input values along each input dimension^2^. However, this problem is alleviated when chunks are created in a more conservative manner. For example, Veksler et al. (2014) employed the ACT-R (Anderson, 1993; Anderson and Lebiere, 1998) rational memory activation mechanism, where memory activation is based on its recency/frequency of use, as a selection mechanism for which memory nodes could be chunked.

In Experiment 1 below we gather data from participants classifying threats in an IDS-like environment, so as to examine how SDL-based cognitive models and DL-based behavior models may be able to learn from smallish data sets of expert behavior in such an environment, and to what degree these methods may be helpful in highlighting alerts for non-expert participants. Specifically, for the simulations below we employ a popular DL framework TensorFlow (Abadi et al., 2016) and the conservative-rational SDL framework (Veksler et al., 2014). The conservative-rational framework was originally proposed as an amalgamation of two other models of symbolic hierarchical memory—the configural-cue memory structure (Gluck and Bower, 1988), and the ACT-R cognitive architecture chunk activation mechanism (Anderson, 1993; Anderson and Lebiere, 1998)—for the purposes of combining the category-learning abilities of the configural-cue model and the computational efficiency of rational memory activation in ACT-R.

The purpose of the simulations below is to provide evidence that the SDL cognitive modeling technique may be useful in the context of aiding security analysts, rather than to find optimal model performance. Thus, we did not perform any parameter search for SDL, and merely used default framework parameters. For DL we attempted simulations with a few different network shapes, so as to establish a more fair comparison of DL and SDL, because network shape is of a very high importance to DL modeling (not so for SDL, since its shape changes automatically). We found that a five-layer network of the shape {*input*, 50, 100, 100, *output*} performed better than networks of smaller or greater depth and networks of smaller or greater widths^3^.

We also attempted different numbers of training epochs for both DL and SDL. Employing multiple training epochs is of high importance for DL when working with smaller datasets. Essentially, if you have 1,000 training samples, training the network for 100 epochs enables you to simulate a dataset of 100,000 samples. Using an overly high number of epochs comes at a cost of overfitting. That is, the model might begin to perform very well on the training data, but will fail to generalize to a new dataset (test data) if the number of epochs is overly high. This is not as important for SDL, as it requires less than ten epochs to reach peak performance even on small datasets, but it is important nonetheless. All simulation results reported below are based on best-performing numbers of epochs for each model.

All simulation results below are averaged over one hundred simulation runs.

### 2.1. Experiment 1

Experiment 1 was designed as a part of a larger study (unpublished) to examine human ability to evaluate cyber-threats in a simplified IDS-like environment based on a small set of instructions. In this experiment participants were presented with four batches of cyber activity records, where Batch 1 had 40 records, Batch 2 had 60 records, Batch 3 had 80 records, and Batch 4 had 100 records. Batch order was randomized. Each record consisted of four features relating to the detected network activity: time stamp, source port number, country, and alert description (e.g., “FTP - Suspicious MGET Command,” “ET TROJAN Qhosts Trojan Check-in”).

For each such record participants were able to click either “Threat” or “No Threat” radio button. Half of the records in each batch were threats. Participants were not provided feedback as to whether their threat classifications were correct, however, threat classification rules (see Table 1) were always visible to the participants.

**Table 1:** Experiment 1 threat classification rules.

**Events are threats if they meet all four criteria:**
1. Time Stamp between 0:00 and 5:00 h
2. Source Port <80 or > 5,000
3. Destination Countries: Russia, China
4. Alerts including suspicious, encrypted, exploit, and virus

This study was performed online, using Amazon Mechanical Turk to recruit adult residents of the United States for pay. We recruited sixty one participants for this experiment.

#### 2.1.1. Results

For the purposes of all model analyses below we employ Batches 1, 2, and 3 as model learning data (i.e., training sets), and Batch 4 (containing 100 record cases) for examination (i.e., test set). The highest overall identification score for Batches 1, 2, and 3 was 0.883, and this score was achieved by four participants. We classify these four participants as “experts” and train SDL and DL models only on those participants' decisions from Batches 1, 2, and 3 (not Batch 4).

Average overall score on Batch 4 for non-expert participants was 71.3% (random-level behavior is 50%), with average hitrate (HR; correctly identified threats) being 0.725 and false-alarm rate (FA) being 0.298^4^. According to Signal Detection Theory (Swets, 1964, 1996), given the same training time we could have pushed participants toward a higher hitrate at the cost of increasing false-alarms or a lower false-alarm rate at the cost of a lower hitrate, depending on the perceived subjective utilities of hits, misses, false alarms, and correct rejections, though what would remain constant is their ability to discriminate what constitutes a threat – the sensitivity characteristic, *d*′. In this experiment the non-expert sensitivity on Batch 4 was *d*′ = 1.128 (higher *d*′ suggests higher sensitivity; *d*′ for random-level behavior is 0).

**Figure 2:**
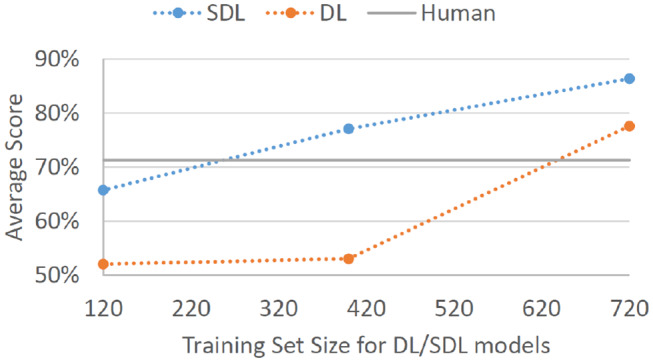
Average correct classification score for SDL and DL models on Batch 4. Models were trained on Batch 1 of expert data (4 experts × 40 decisions each = 120 total cases), Batches 1 and 2 of expert data (4 experts × 100 decisions each = 400 total cases), and Batches 1, 2, and 3 of expert data (4 experts × 180 decisions each = 720 total cases). Gray baseline labeled “Human” represents average performance on Batch 4 for human non-expert participants.

SDL model trained^5^ on expert decisions from Batches 1, 2, and 3 (180 cases × 4 experts) produced an average score of 86.4% on Batch 4, with average hitrate being 0.796 and average false-alarm rate being 0.069, *d*′ = 2.31. DL model trained^6^ on expert decisions from Batches 1, 2, and 3 produced an average score of 77.6% on Batch 4, with average hitrate being 0.701 and average false-alarm rate being 0.149, *d*′ = 1.57. When trained on just Batches 1 and 2 (100 cases × 4 experts) SDL^7^ and DL^8^ *d*′ statistics fell to 1.76 and 0.16, respectively. When trained on just Batch 1 (40 cases × 4 experts) SDL^9^ and DL^10^ *d*′ statistics fell to 0.84 and 0.14, respectively. Figure 2 displays the overall performance scores for SDL and DL given the three training set sizes.

**Figure 3:**
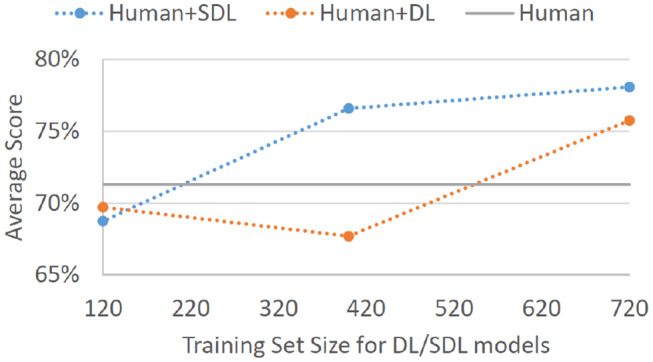
Average correct classification score for non-experts on Batch 4 with the assumption that the non-experts would adopt all threat suggestions provided by a given helper-agent. The displayed results are for SDL and DL helper-agents that were trained on Batch 1 of expert data (4 experts × 40 decisions each = 120 total cases), Batches 1 and 2 of expert data (4 experts × 100 decisions each = 400 total cases), and Batches 1, 2, and 3 of expert data (4 experts × 180 decisions each = 720 total cases). Gray baseline labeled “Human” represents average performance on Batch 4 for non-expert participants without any helper-agent suggestions.

Perhaps more important than a standalone model score on Batch 4 is the degree to which such models can aid non-experts in their decision-making. Assuming that we employ SDL trained on expert decisions from Batches 1, 2, and 3 to highlight potential threats on Batch 4, and assuming that non-expert participants always classified the highlighted records as threats, non-expert hitrate would go up to 90.4%. This is a 25% improvement on correctly identified threats. This would come at a cost of false alarms rate increasing to 34.3% (15% increase); however, the overall ability to discriminate signal from noise for such human-agent teams would go from *d*′ = 1.13 to *d*′ = 1.71. Even if SDL was trained only on 100 decisions from each of the four experts (just Batches 1 and 2), the overall sensitivity to the threat signal would improve, *d*′ = 1.54 (*HR* =.870, *FA* = 0.338). Employing SDL trained only on Batch 1 (40 decision from each of the four experts) would provide no decision improvement, *d*′ = 1.12 (*HR* =.861, *FA* = 0.486). As would be expected based on standalone model performances, an analogous DL-based decision could help humans improve to a slightly lesser degree when trained on expert decisions from Batches 1, 2, and 3, *d*′ = 1.62 (*HR* = 0.913, *FA* = 0.397), and not at all when trained only on Batches 1 and 2, *d*′ = 0.98 (*HR* = 0.801, *FA* = 0.447), or only on Batch 1, *d*′ = 1.05 (*HR* = 0.767, *FA* = 0.372). Figure 3 displays the overall performance scores that can be achieved for non-expert human-agent teams given different sizes of expert decision training sets.

### 2.2. Discussion

One of the most clear results above is that SDL performs much better with smaller datasets than traditional DL methods. This matters a great deal in the field of cybersecurity, where expert data is difficult to come by, or where expertise is localized to a single 12-hour shift. Perhaps it should not be surprising that cognitive modeling methodology is more appropriate for building decision aids based on small samples of individual decisions than AI methods designed for large data mathematical optimization. Unfortunately, due to the popularity of traditional ML methodology, cognitive computing is often not taken into account, even when it may be the right tool for the job.

Note that the expert decisions that models were trained on were only 88.3% correct and SDL was able to achieve nearly this same performance, 86.4%, on the 100 test cases. If non-experts were to have complete trust that records highlighted as threats by the SDL decision aid are correct, their performance could improve from 71.3 to 78.1%. We could presume that if expert performance was better, and if more expert data was available, SDL performance and the degree to which it could help non-experts would improve, as well. On the flip side, if expert performance was worse, or if less expert data were available, we would expect worse performance from both SDL and DL. This suggests that experiments of this ilk may not replicate with smaller samples of participants. More importantly, selection of expert performers in the real world is of the highest concern for generating similar decision-aid training data.

One question that may come to mind is whether humans are needed at all. If it is the case that SDL performance is 86.4% whereas non-expert human performance is expected to be between 71.3 and 78.1% even with the SDL-based decision-aid, why not just train agents on expert behavior and let them loose without non-expert interference? However, this questions presumes static non-expert performance, whereas humans learn and adapt. Human novices may begin with lower levels of performance, but when provided with expert feedback, their performance improves. Cognitive modeling -based decision support isn't meant to supplant non-experts, but rather to give them immediate expert-based feedback, so as to help them make better decisions in the early trials, and reach expert-level performance at a faster rate than otherwise would have happened.

Moreover, the projected proportion of missed threats for the human-agent team, 9.6%, is lower than it would be either for the non-expert humans, 17.5%, or for standalone SDL, 20.4%. Thus, if we cared more about missed threats than false alarms, as is often the case in cyber, human-agent teaming is the ultimate option in this paradigm.

To be clear, SDL missed-threat rate can be decreased at the cost of a higher false alarm rate via a different reward structure (it is the case that the conservative-rational SDL framework includes a reinforcement learning component that is sensitive to the reward structure). However, if it was the case that the human analyst had a high degree of trust in SDL-based alert highlights, the human-agent team elevated alerts would be a superset^11^ of those that would have been elevated by the human and a superset of a large proportion of SDL-elevated alerts.

We would be remiss not to point out that a decision aid will cease to be helpful without a degree of trust from the human analyst. We project that SDL-generated cognitive models of expert analysts will impart a high degree of trust for at least two reasons – (1) model-based suggestions promise to greatly improve overall non-expert performance, and (2) according to Veksler and Buchler (2019), SDL promises to be a more transparent technique than DL, one that is able to provide some explainability for each of its suggestions. The full extent to which such performance improvement and transparency may aid in establishing trust with human participants remains a topic for future research.

Overall, we find these results promising, and argue strongly that cognitive modeling can be highly useful for learning from expert analyst preferences and simulating expert-like decisions in the context of cyber support or training.

## 3. Dynamic Cognitive Models of Attacker Preferences

Cybersecurity is, at its core, a fundamentally adversarial paradigm. It comprises a repeated cycle where cyber defense specialists attempt to predict potential attack paths, and a cyber attacker attempts to pick an attack path to overcome the potential defense strategies. This formalism lends itself well to Game Theory (GT) -based approaches for repeated security games.

Indeed, GT-based software has been successfully applied in real-world security contexts, including airport security, coast guard, police, and anti-poaching (animal preservation) efforts (Tambe et al., 2014), providing much-needed evidence that theory based on small toy problems scales to real-world asymmetric^12^ security contexts. Real world security decision aids go beyond normative game theory (picking some optimal mix of actions assuming a perfectly rational opponent), and attempt to include attacker *subjective* utilities in the equation. Recent research has shown that GT approaches to defense can be improved by relaxing the assumption of human optimal behavior and updating assumed attacker subjective utilities based on known attacker actions and feedback (Abbasi et al., 2015; Kar et al., 2015; Cooney et al., 2019; Cranford et al., 2019).

Veksler and Buchler (2016) provide simulation predictions showing that cognitive modeling -based approaches can thwart 10–50% more attacks than normative GT approaches. Specifically, they describe how Reinforcement Learning (RL)-type models may be tuned to individual attacker's subjective preferences and learning abilities via *model tracing* and *dynamic parameter fitting*. The model tracing technique makes use of boot-strapping to force-feed the participant's current experiences to the cognitive model. That is, if the participant and the model were to choose different strategies, model actions would be overwritten with participant actions in the model's memory. This method was employed in computerized instructional aids, “cognitive tutors,” for students learning high school math (Anderson et al., 1995). Dynamic parameter fitting is used to adjust model parameters based on known data points, so as to make better individual predictions for future behavior. That is, if there is a free parameter in the model (such as the learning-rate parameter in the Veksler and Buchler, 2016, simulations), a range of values for this parameter are plugged into the model, and the value that best fits individual's recorded behavior is then retained for predicting their future behavior. This method was employed to predict performance of individual F-16 pilot teams (Jastrzembski et al., 2009) and is employed in software that predicts optimal training schedules based on individual performance histories (Jastrzembski et al., 2014).

The RL model used in the Veksler and Buchler (2016) simulations, as well as in the experiments described below, is based on the ACT-R utility learning mechanism (Fu and Anderson, 2006; Anderson, 2007). The model-tracing RL assumes a human attacker's action preferences will change based on their experience. For example, if the attacker chooses *A*1 and happens to lose, they will be less likely to choose *A*1 in future attacks, regardless of whether *A*1 is ultimately a good choice. Conversely, if the attacker chooses *A*1 and happens to win, they will be more likely to choose *A*1 in future attacks, regardless of whether *A*1 is ultimately a poor choice. More formally, after performing some action, *A*, the expected utility of this action, *U*_*A*_, is incremented by the following term:

(1) $$\begin{array}{l} \Delta U_{A}=\alpha \left(R-U_{A}\right), \end{array}$$

where α is the learning rate, and *R* is the value of the feedback (e.g., success/failure, reward/punishment).

Experiments 2a and 2b below attempt to validate Veksler and Buchler (2016) predictions and provide an in-depth analysis as to overall and individual effectiveness of using predictive models to pick strategies against human attackers. The simulations employed an abstract security game paradigm where the defender and attacker each had four potential strategies to choose from (payoff table showing attack success probabilities displayed as **Table 3**). Although the security game setup is abstract enough that it can fit any security context^13^, we would argue that it is particularly relevant in the context of cyber security, as this is a domain where the state of the task-environment and human actions are immediately recordable.

### 3.1. Experiment 2a

Experiment 2a was designed to validate Veksler and Buchler (2016) simulation predictions for how the use of model tracing (MT) and model tracing in combination with dynamic parameter fitting (MT++) can improve upon game theory-based Fixed-strategy^14^ and optimal Mixed-strategy^15^ approaches. That is, this experiment comprised a repeated game scenario where each human participant played the role of an attacker, playing against some computational agent defender. The four between-subject conditions in this experiment corresponded to four types of computational agent defenders that human attackers were playing against: Fixed, Mixed, MT, and MT++.

This study was performed online, using Amazon Mechanical Turk to recruit adult residents of the United States for pay. We recruited 40 participants per condition.

Participants were payed 50 cents, plus five cents per win (maximum total of $3.00), and were notified as to this pay structure prior to the study. Experiment instructions were randomly altered to employ one of two contexts corresponding to cybersecurity and physical security games (see Table 2).

**Table 2:** Experiment 2 instructions.

**Cyber game**	**IED game**
In this study you will play multiple rounds of the Cyber game. The game has two sides the Blue Force and the Red Force. The Blue Force aims to protect sensitive data. The Red Force aims to hack into the Blue Force computer network and steal the protected data. The Blue Force player will be controlled by the computer. In each round Blue Force will pick its strategy regarding which parts of the network to scan for intrusions, and how to perform those scans. You are assigned the role of Commander of the Red Force. In each round you will make 2 choices to select Red Force strategy: • Whether to focus your team's attack on the main server, or to distribute the attack over multiple servers. • Whether to scan for vulnerabilities intermittently (safer, less likely that the scan will be detected), or to scan continuously (faster). At the end of each round you will be notified whether you win the round (i.e., data acquired), or lose the round (i.e., no data was acquired).	In this study you will play multiple rounds of the IED game. The game has two sides the Blue Force and the Red Force. The Blue Force aims to deliver aid to a village. The Red Force aims to block the Blue Force form getting to the village by planting Improvised Explosive Devices (IEDs) along village routes. The Blue Force player will be controlled by the computer. In each round Blue Force will pick its strategy regarding which roads to use to get to the village, and whether to deliver aid fast, or to use more caution. You are assigned the role of Commander of the Red Force. In each round you will make 2 choices to select Red Force strategy: • Whether to plant all IEDs along the main road, or split your IEDs up and plant them along multiple roads leading into your village. • Whether to use stealth movements (safer), or to go without stealth (faster). At the end of each round you will be notified whether you win the round (i.e., Blue Force is defeated), or lose the round (i.e., Blue Force succeeds in their mission).

Each human participant played 50 games (i.e., trials) against their respective opponent. On each trial participant made two binary choices (see experiment instructions in Table 2), thus choosing among four potential attack types for that game. The computational agent also chose among four potential actions. Just as in the predictive simulations described by Veksler and Buchler (2016), the probability of a successful attack was based on the strategy selections of both players, as shown in Table 3. After a participant made their choices, they were alerted as to whether the attack was successful or not, and then the next game instance began.

**Table 3:** Payoffs for the attacker (probability of successful attack) in a security game used for Veksler and Buchler (2016) simulation predictions, as well as in Experiments 2a and 2b.

		**Attacker**			
		**A1**	**A2**	**A3**	**A4**
Defender
	D1	0.15	0.45	0.50	0.90
	D2	0.55	0.10	0.90	0.45
	D3	0.50	0.90	0.15	0.45
	D4	0.90	0.50	0.50	0.10

*There are four possible actions for the defender {D1,D2,D3,D4}, and four possible actions for the attacker {A1,A2,A3,A4}*.

#### 3.1.1. Results

**Figure 4:**
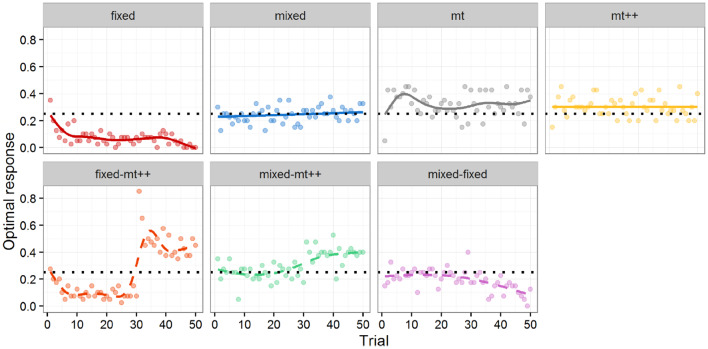
Optimal response by trial for computational agent. Markers represent percentage of optimal response per trial. Lines represent a LOESS curve fit on data from individual participants. Solid lines **(top)** are from Experiment 2a and dashed lines **(bottom)** from Experiment 2b. Experiment 2b results all include a shift (i.e., a switch) from one type of opponent to another after 30 trials (e.g., in the *fixed-mt*++ condition, human participants play against a Fixed-strategy agent for 30 games, and then against MT++ agent for the last 20 games). Dotted horizontal line indicates expected performance from random play.

The top of Figure 4 shows the performance of the different computational agents against the human attackers from Experiment 2a over the 50 trials. Performance is measured by whether or not the computational agent selected the response that maximized its probability of winning against the attacker, referred to as the optimal response^16^. As there are four possible responses, and a single response that maximizes the probability of winning, random play would result in selecting the optimal response 25% of the time.

To test for differences between the computational agent strategies and random play, we ran a mixed effect logistic regression using whether the computational agent selected an optimal response as the dependent variable, the type of computational agent as fixed effects, and the participant against which it played as random effects, with a fixed intercept of log(1/3), i.e., 25%. The logistic regression addresses the binary nature of our outcome measure; the random effects account for the multiple measures coming from the same participant; and the fixed intercept provides comparisons with our baseline of interest (random play). Although both computer and human agents may learn over time, the regression focuses on “aggregate” performance over the 50 trials and does not include a covariate for trial. The fixed strategy does significantly worse than random play, whereas both model tracer strategies do significantly better than random play. As the mixed strategy is effectively random, it did not significantly differ from the random play baseline, as expected.

**Figure 5:**
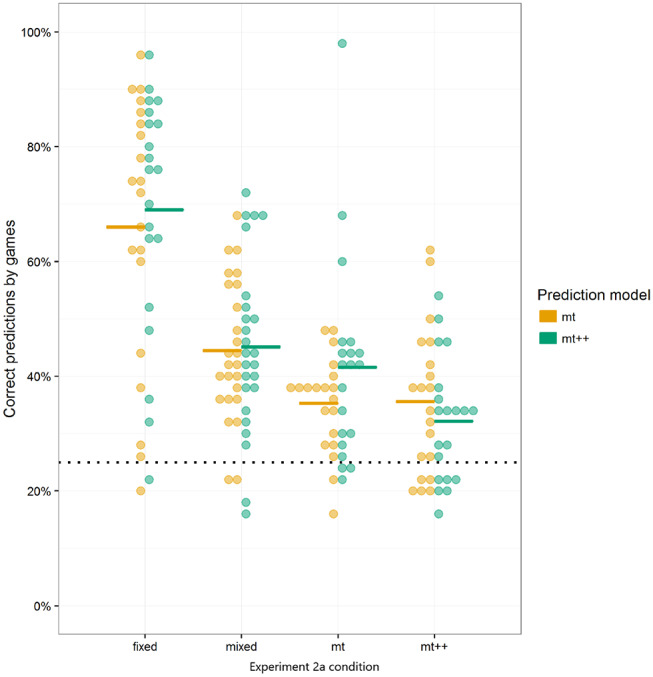
MT and MT++ *post-hoc* predictions of participant behavior across all 4 treatments. Dots represent individual participants and dashes represent averages. Horizontal dotted lines represent expected percent of correct predictions from random guesses.

While both model tracer strategies did better than random play, the effect sizes (approx. +7 percentage points for MT, and +5 percentage points for MT++) were relatively modest. We speculate (and test this speculation in Experiment 2b) that participants may have been able to adapt to the model tracer strategies, learning to be more “unpredictable.” In *post-hoc* analysis, we evaluated how well MT and MT++ models could predict human decisions across all four treatments. Both MT and MT++ models appear better at predicting participant behavior in the fixed and mixed conditions than in the model tracing conditions (see Figure 5). There also appears to be a potential interaction in that each model tracer may be better at predicting behavior in the condition for the *other* model tracer (MT++ predicts a higher rate of decisions where human participants are playing against MT than against MT++, and MT predicts a higher rate of decisions where human participants are playing against MT++ than against MT). In combination, these findings are consistent with participants adapting to the model tracer agents, making themselves less predictable.

**Figure 6:**
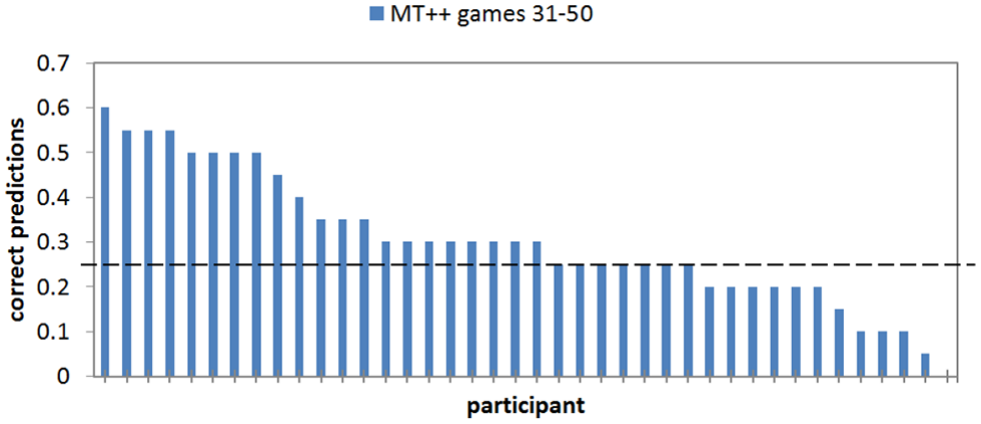
Proportion of correct predictions of individual human participant choices in the last 20 games of the MT++ condition. Horizontal dashed line represent expected percent of correct predictions from random guesses.

In analyzing the last 20 trials of the MT++ condition we find that 40–60% of the decisions were still predictable for ten out of the 40 participants (25%); 25 of the remaining participants were predictable 15–35% of the time (25% is chance), and the final five participants in this condition were predictable for ≤ 10% of their last 20 decisions (see Figure 6). The chances of the predicted choice being avoided 20 times in a row, as one of the participants managed to do, are about 3:1,000. The indication is that these individuals can predict what the predictive agent will predict, and do the opposite. Thus, it seems that keeping predictive abilities hidden until some critical juncture would increase model ability to thwart attacks at said juncture. Experiment 2b examines this hypothesis.

### 3.2. Experiment 2b

Experiment 2a results suggest that human opponent decisions are easier to predict when said opponent does not know that they are playing against a predictive agent. In other words, if we can predict attacker actions, but then withhold this predictive ability, we can achieve high levels of success at some later critical point in time. Experiment 2b was designed to validate this hypothesis.

The methods in Experiment 2b are the same as those in Experiment 2a, with the exception of defender agent types. Specifically, this study includes three conditions, corresponding to three new agent types. The first of the agents is the MT++ agent from Experiment 2a, but it plays a fixed strategy for the first 30 of the 50 games (Fixed-MT++). The second of the agents is the MT++ agent from Experiment 2a, but it plays a mixed strategy for the first 30 of the 50 games (Mixed-MT++). The third agent, added as a control condition, plays a mixed strategy for the first 30 games, and plays a fixed strategy for the remaining 20 (Mixed-Fixed).

#### 3.2.1. Results

The bottom of Figure 4 shows the performance of the new multiple strategy computational agents against the human attackers from Experiment 2b over the 50 trials. As in Experiment 2a, performance is measured by optimal response.

To test for differences between the computational agents and random play and to look at performance across the distinct strategy phases, we ran a mixed effect logistic regression using optimal response as the dependent variable, the type of computational agent interacted with whether the trials were in the first segment (first 30 trials) or in the second segment (last 20 trials) of the game as fixed effects, and the participant as random effects, with a fixed intercept of log(1/3). We reproduce our results from Experiment 2a in both portions of the game for each strategy—finding that the fixed strategy is significantly less likely to select the optimal response, and both model-tracing strategies are significantly more likely to select the optimal response relative to random play in both portions of the game. The mixed strategy is not significantly different from random play. In addition, in our multiple strategy agents, we find that the Fixed-MT++ agent is significantly less likely to select the optimal response in the first 30, and more likely to select the optimal response in the last 20 compared to random play; the Mixed-MT++ is not significantly different from random play in the first 30, but significantly more likely to select the optimal response in the last 20; and the Mixed-Fixed is not significantly different from random play in the first 30, but significantly less likely to select the optimal response in the last 20.

To look more closely at how performance changes in the multiple strategy agents, and to compare the multiple strategy MT++ agents to baselines, we (1) test contrasts of agent performance between the first and second segments of the multiple strategy agents, and (2) test contrasts of MT++ performance in the last 20 trials of the multiple strategy MT++ agents and MT++ performance in the first 30 and last 20 trials of the MT++ strategy from Experiment 2a. These contrasts are tested as a single family using the mvt adjustment from the R lsmeans package.

In terms of performance changes, results confirm that between the first 30 and the last 20 trials, performance significantly increases in the Fixed-MT++ agent (Figure 4 bottom-row, left), *p* < 0.001; significantly increases in the Mixed-MT++ agent (Figure 4 bottom-row, middle), *p* < 0.001; and significantly decreases in the Mixed-Fixed agent (Figure 4 bottom-row, right), *p* < 0.001.

In terms of relative performance, we find that when the Fixed-MT++ agent shifts to MT++ in the last 20 trials, it outperforms the normal MT++ agent in both its first 30 trials, *p* = < 0.001, and its last 20 trials, *p* < 0.001; and outperforms the Mixed-MT++ agent in the last 20 trials, *p* = 0.048. When the Mixed-MT++ agent shifts to MT++ for the last 20 trials, it outperforms the normal MT++ agent in both its first 30 trials, *p* = 0.006, and the last 20 trials, *p* = 0.008.

**Figure 7:**
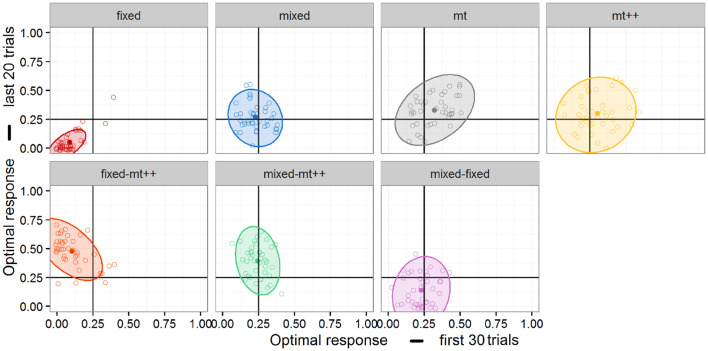
Individual performance in first and second segments by each agent. Ellipses represent 95% confidence.

In conjunction with the analysis of changes in performance, individual performance in the first and second segments of each game was plotted in Figure 7. *Post-hoc* exploratory analysis suggests additional differences between the performance of the strategies. Notably, we see a positive correlation between first and second segment behavior in the Fixed and MT agents, which suggests an element of “skill” – either on the part of the human player or the computational agent. In contrast, neither the Mixed nor MT++ agents show any correlation, meaning that people (and computational agents) that perform relatively better in the first segment are no more likely to perform better in the second segment. This suggests that, while the MT and MT++ strategies may have similar optimal response rates as suggested by Experiment 2a, there are nonetheless differences in the way these strategies interact with their opponents.

A second relationship of interest can be noted in the second row of Figure 7. In particular, the Fixed-MT++ agent shows a negative correlation between first and second segments whereas the Mixed-MT++ shows a weak one—if any. One possible interpretation of this negative correlation is that, the switching strategy may be particularly effective if the opponents are able to adopt some counter-strategy in the initial trials.

**Figure 8:**
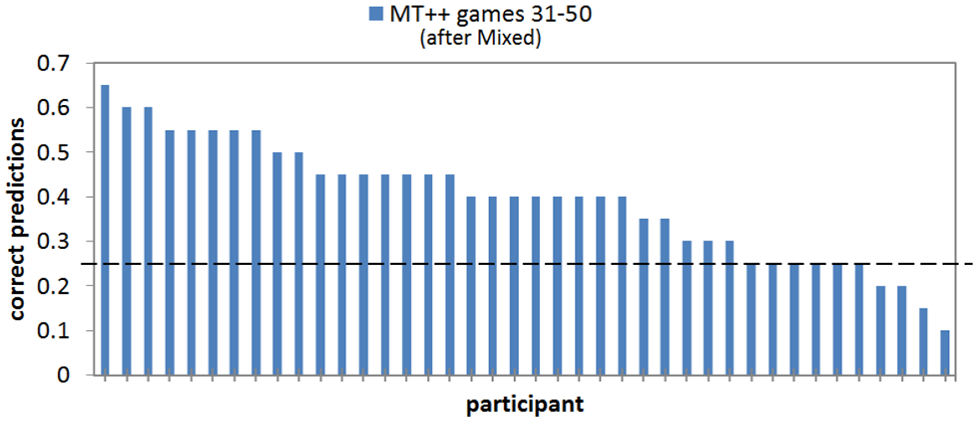
Proportion of correct predictions of individual human participant choices in the last 20 games of the MT++ condition. Horizontal dashed line represents expected proportion of correct predictions from random guesses.

**Figure 9:**
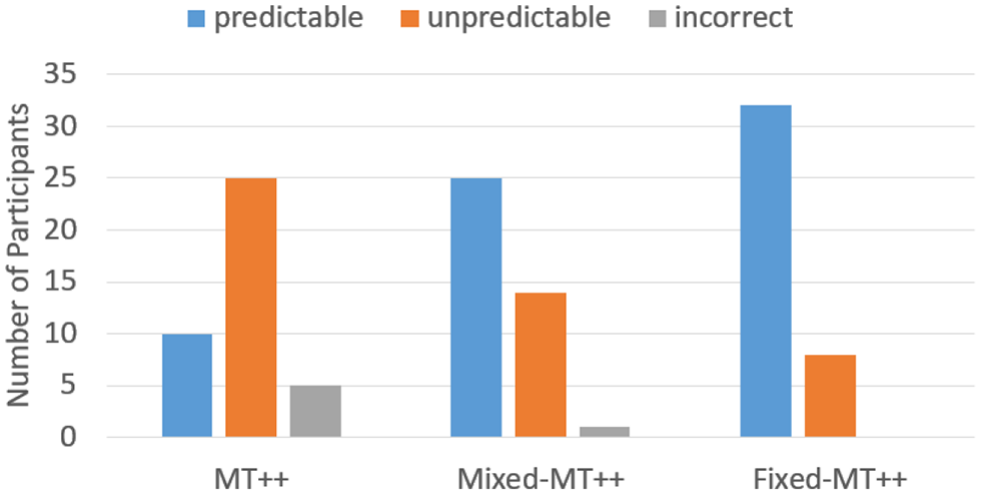
Number of participants whose choices were predictable, unpredictable, or incorrect playing against MT++ in the last 20 trials of the experiment. Participants were labeled as *predictable* if more than 40% of their decisions were correctly predicted by the model, *unpredictable* if their decisions were predicted between 15 and 35% (25% is chance), and *incorrect* if their decisions were the opposite of what was predicted.

When we break down the results by individual predictability we find that keeping predictive abilities hidden greatly mitigates attacker ability to adapt and become less predictable. For example, in the last 20 games of the Mixed-MT++ condition, 40–65% of the choices were predicted for 25 of the participants (62.5%), 15–35% of the choices were predicted for fourteen of the participants, and only one individual was predictable on 10% of their choices (see Figure 8). The results from the last 20 trials of the Fixed-MT++ condition are better still, with 32 individuals being predictable at 40–70%, and the remaining 8 at 20–35%. The overall predictability of attackers playing against MT++ the entire time (from Experiment 2a) and MT++ after switching from Mixed and Fixed strategies are shown in Figure 9.

### 3.3. Discussion

Results from these experiments confirm the general prediction that cognitive modeling techniques can be more effective than normative GT in the context of predicting attacker decisions. However, the average advantage of cognitive modeling over GT seems to be greatly diminished when attackers realize they are playing against a predictive agent. That is, when human players know that they are matched against a predictive agent, their play changes and becomes less predictable. However, this does not mean that all participants learn to play [pseudo-]randomly against predictive agents. Rather, some individual game-play remained predictable, some looked more like chance play, and some of the individuals began to predict the predictive agent, adopting a “Theory of Mind” (ToM) strategy^17^. The less “smart” of an agent human participants were matched against, the more predictable their play became, with participants that played against a Fixed-strategy agent becoming the most predictable, those playing against a Mixed-strategy agent being less so, and those playing against MT++ being the least predictable.

Ultimately, by keeping track of prediction success for each individual attacker, a defender agent should be able to ascribe the correct model to the attacker: random play, RL, or ToM. Once the correct model of the attacker is determined, the defender can choose its own appropriate strategy: Mixed strategy, a predictive strategy, or the opposite of the predictive strategy, respectively. Seemingly, once the human attacker realizes that they are playing against a predictive agent and switches to a reciprocal strategy, and the agent switches its strategy in turn, the two opponents may continue to switch their game-play continuously. However, it is not the case that this would necessarily end with Mixed strategy level of play by the two opponents, as humans are notoriously bad at being random. West and Lebiere (2001) predict that chaos-like game-play may actually be an emergent property of reciprocal and predictable human choices.

This paper only explores standalone RL as a potential cognitive model for predicting attacker choices. More sophisticated attacker models, including those based on ToM strategies, the work of West and Lebiere (2001), and models that include domain-specific knowledge, should be able to account for a greater range of attacker behavior. Attacker models can be further seeded based on types of attacker personalities, risk-tolerance, and attack-types common to specific geographic regions (e.g., Sample, 2015). Having a greater wealth of model types would be a major boon to dynamically fitting individual attacker behavior, and would result in more precise and accurate predictions of further attacker choice. In a more general sense, we argue that Cognitive Modeling as a discipline is useful for predicting individual preferences and behavior, and is thus highly relevant for real-time cybersecurity decision support.

## 4. Summary

Prior work has argued that cognitive modeling techniques can be trained on expert data so as to provide such expertise as an aid for non-experts, and that CM-based Symbolic Deep Learning would be more useful in this endeavor than ML-based Deep Learning frameworks, especially in fields like cybersecurity where expert data is not highly abundant (Veksler and Buchler, 2019). In a similar vein, other work has made strong predictions that cognitive modeling may be useful in predicting opponent decision preferences in repeated security games, and be more useful than normative GT-based security aids, especially in fields like cybersecurity where behavior/feedback of attackers can be dynamically observed/updated (Veksler and Buchler, 2016). We presented Experiments 1 and 2 above so as to examine these predictions against human data.

Experiment 1 results revealed that CM-based SDL framework is more effective than ML-based DL framework in learning from experts and has much more potential for improving non-expert performance. The separation between SDL and DL effectiveness greatly increases as the available training data gets more sparse. Regardless of model type or training data, it is the case that a human-agent team where the human non-expert always accepts model suggestions for elevating alerts will have a higher alert hitrate than either a lone human or a lone model. Future work in this domain will focus on the topic of trust, and examining the degree to which SDL-based decision-aids will be trusted by human non-experts. We project that the overall level of performance improvement and the potential for decision-explainability that SDL-generated cognitive models can provide will create enough trust to develop highly effective teams of human-agent analysts.

Experiment 2 results revealed that model-tracing and dynamic parameter-fitting techniques can be used to continuously update cognitive models of attackers and to accurately predict a high percentage of their decisions. Results further indicate that when model predictive capabilities are hidden from the opponent, the opponent's decisions become more predictable, especially when said opponent believes they are playing against an unsophisticated defender. Our conclusion is that in adversarial repeated cybersecurity contexts cognitive models should be tuned to individual attacker's preferences, but model predictive abilities should be held hidden until some critical juncture so as to maximize effect. Future work will focus on development of more sophisticated models, such that when attackers recognize a model's predictive abilities and attempt to pivot to unforeseen strategies, the model can make a timely pivot, as well.

The experiment and simulation results presented here look promising. However, this paper presents theoretical models examined in absence of real-world confounds. Future work will focus on stress-testing these models in the context of real cybersecurity data. Although it is the case that “[t]here is nothing so useful as a good theory,” (Lewin, 1951, as cited by Gray and Altmann, 2001) it is also the case that “[n]othing drives basic science better than a good applied problem” (Newell and Card, 1985, as cited by Gray and Altmann, 2001). We believe that the methods presented in this paper can be of great use for cybersecurity, but also that the applied problem of cybersecurity itself and the datasets derived in this domain can serve to refine these methods and to push them from research stages and toward production.

On a more general note, we would argue that Cognitive Science, and specifically Cognitive Modeling as a discipline, is highly relevant and holds great promise in cybersecurity and analogous domains. Models of human cognition can be automatically tuned to either defender or attacker preferences, and such models can then be used in simulations, training, and decision aids. Whereas network/software vulnerabilities change constantly, the fundamentals of human learning and decision-making principles remain the same. In taking advantage of established and emerging cognitive and behavioral research and technology we can vastly improve our overall long-term network safety.

## Data Availability Statement

The datasets generated for this study are available on request to the corresponding author.

## Ethics Statement

The studies involving human participants were reviewed and approved by U.S. Army Research Laboratory. The patients/participants provided their written informed consent to participate in this study.

## Author Contributions

All authors listed have made a substantial, direct and intellectual contribution to the work, and approved it for publication.

## Conflict of Interest

VV was employed by the company DCS Corp. The remaining authors declare that the research was conducted in the absence of any commercial or financial relationships that could be construed as a potential conflict of interest.

## Footnotes

^1^Model-tracing comprises force-feeding individual human experiences into the model. More on this in Dynamic Cognitive Models of Attacker Preferences section.

^2^Given *n* features (e.g., *large*, *square*, *white*), we can create a chunk for every combination of feature presence and absence ({*large*}, {*square*}, {*white*}, {*large, square*}, {*large, white*}, {*square, white*}, and {*large, square, white*}). If we represent feature presence as a 1 and feature absence as a 0, we can represent each chunk as a binary number, and the total number of possible chunks is the total number of possible binary numbers, minus the blank chunk, which is 2^*n*^−1. When each feature dimension can have two potential values, the total number of possible chunks is 3^*n*^−1. With *k* possible values on *n* feature dimensions, we can have at most (*k*+1)^*n*^−1 possible chunks to represent all potential feature combinations.

^3^All performance comparisons were in terms of the sensitivity heuristic, *d*′. Network layer depths were incremented and decremented by one layer for comparisons. Network layer widths were incremented and decremented by 25–50 nodes for comparisons.

^4^All analyses include only Batch 4 cases where participants made a threat/no-threat classification. Not all presented cases were answered by all participants. Forty nine participants provided all 100 answers, four of the participants provided only 99 answers, one participants provided only 77 answers, and three other participants provided <20 answers each.

^5^3 epochs.

^6^100 epochs.

^7^6 epochs.

^8^200 epochs.

^9^8 epochs.

^10^200 epochs.

^11^Set A is a superset of B, or equivalently set B is a subset of A, if B is contained in A.

^12^Attacker-defender dynamics are naturally asymmetric, where the attacker is much less limited in their methods than the defender in their countermeasures.

^13^In the experiments below we employed both cyber and physical security contexts, and found no significant difference in performance between the two contexts, *p* > 0.2.

^14^In game theory a fixed strategy— always employing the same action path—is often employed to establish a baseline level of performance.

^15^In game theory an optimal mixed strategy is one where strategies are selected randomly from a specific distribution that is tuned so as to leave the opponent no preferred choices.

^16^Maximizing the probability of winning is actually what the defender agents are designed to do; so optimal response is, in effect, a direct measure of success for the defender agent. Of course, when different attacker action paths are aimed at different targets of unequal value, optimal response is one that maximizes the likelihood of preventing an attack weighed by target value.

^17^The concept of Theory of Mind refers to one's ability to infer others' beliefs and intentions (e.g., Hiatt and Trafton, 2010).
